# Patterns of patient-reported symptoms and association with sociodemographic and systemic sclerosis disease characteristics: a scleroderma Patient-centered Intervention Network (SPIN) Cohort cross-sectional study

**DOI:** 10.1016/j.eclinm.2023.102104

**Published:** 2023-07-20

**Authors:** Robyn K. Wojeck, Mitchell R. Knisely, Donald E. Bailey, Tamara J. Somers, Linda Kwakkenbos, Marie-Eve Carrier, Warren R. Nielson, Susan J. Bartlett, Vanessa L. Malcarne, Marie Hudson, Brooke Levis, Andrea Benedetti, Luc Mouthon, Brett D. Thombs, Susan G. Silva, SPIN Investigators, Claire E. Adams, Richard S. Henry, Catherine Fortuné, Karen Gottesman, Geneviève Guillot, Laura K. Hummers, Amanda Lawrie-Jones, Maureen D. Mayes, Michelle Richard, Maureen Sauvé, Shervin Assassi, Ghassan El-Baalbaki, Kim Fligelstone, Tracy Frech, Amy Gietzen, Daphna Harel, Monique Hinchcliff, Sindhu R. Johnson, Maggie Larche, Catarina Leite, Christelle Nguyen, Karen Nielsen, Janet Pope, François Rannou, Tatiana Sofia Rodriguez-Reyna, Anne A. Schouffoer, Maria E. Suarez-Almazor, Christian Agard, Nassim Ait Abdallah, Marc André, Elana J. Bernstein, Sabine Berthier, Lyne Bissonnette, Alessandra Bruns, Patricia Carreira, Marion Casadevall, Benjamin Chaigne, Lorinda Chung, Benjamin Crichi, Christopher Denton, Robyn Domsic, James V. Dunne, Bertrand Dunogue, Regina Fare, Dominique Farge-Bancel, Paul R. Fortin, Jessica Gordon, Brigitte Granel-Rey, Aurélien Guffroy, Genevieve Gyger, Eric Hachulla, Sabrina Hoa, Alena Ikic, Suzanne Kafaja, Nader Khalidi, Kimberly Lakin, Marc Lambert, David Launay, Yvonne C. Lee, Hélène Maillard, Nancy Maltez, Joanne Manning, Isabelle Marie, Maria Martin Lopez, Thierry Martin, Ariel Masetto, François Maurier, Arsene Mekinian, Sheila Melchor Díaz, Mandana Nikpour, Louis Olagne, Vincent Poindron, Susanna Proudman, Alexis Régent, Sébastien Rivière, David Robinson, Esther Rodríguez Almazar, Sophie Roux, Perrine Smets, Vincent Sobanski, Robert Spiera, Virginia Steen, Evelyn Sutton, Carter Thorne, John Varga, Pearce Wilcox, Mara Cañedo Ayala, Vanessa Cook, Sophie Hu, Bianca Matthews, Elsa-Lynn Nassar, Marieke Alexandra Neyer, Julia Nordlund, Sabrina Provencher

**Affiliations:** a University of Rhode Island, Kingston, Rhode Island, USA; b Duke University School of Nursing, Durham, North Carolina, USA; c Duke University School of Medicine, Durham, North Carolina, USA; d Department of Clinical Psychology, Radboud University, Nijmegen, the Netherlands; e Department of Medical Psychology, Radboud Institute for Health Sciences, Radboud University Medical Center, Nijmegen, the Netherlands; f IQ Healthcare, Radboud Institute for Health Sciences, Radboud University Medical Center, Nijmegen, the Netherlands; g Radboudumc Center for Mindfulness, Department of Psychiatry, Radboud Institute for Health Sciences, Radboud University Medical Center, Nijmegen, the Netherlands; h Lady Davis Institute for Medical Research, Jewish General Hospital, Montreal, Quebec, Canada; i Lawson Health Research Institute, London, Ontario, Canada; j Department of Medicine, McGill University, Montreal, Quebec, Canada; k Research Institute of the McGill University Health Centre, Montreal, Quebec, Canada; l Department of Psychology, San Diego State University, California, USA; m San Diego State University/University of California, San Diego Joint Doctoral Program in Clinical Psychology, San Diego, California, USA; n Department of Epidemiology, Biostatistics, and Occupational Health, McGill University, Montreal, Quebec, Canada; o Respiratory Epidemiology and Clinical Research Unit, McGill University Health Centre, Montreal, Quebec, Canada; p Service de Médecine Interne, Centre de Référence Maladies Autoimmunes Systémiques Rares d'Ile de France, Hôpital Cochin, Assistance Publique-Hôpitaux de Paris (AP-HP), Paris, France; q APHP-CUP, Hôpital Cochin, Université de Paris, Paris, France; r Department of Psychiatry, McGill University, Montreal, Quebec, Canada; s Department of Psychology, McGill University, Montreal, Quebec, Canada; t Biomedical Ethics Unit, McGill University, Montreal, Quebec, Canada

**Keywords:** Systemic sclerosis, Patient-reported symptoms, Symptom cluster

## Abstract

**Background:**

Systemic sclerosis is a heterogenous disease in which little is known about patterns of patient-reported symptom clusters. We aimed to identify classes of individuals with similar anxiety, depression, fatigue, sleep disturbance, and pain symptoms and to evaluate associated sociodemographic and disease-related characteristics.

**Methods:**

This multi-centre cross-sectional study used baseline data from Scleroderma Patient-centered Intervention Network Cohort participants enrolled from 2014 to 2020. Eligible participants completed the PROMIS-29 v2.0 measure. Latent profile analysis was used to identify homogeneous classes of participants based on patterns of anxiety, depression, fatigue, sleep disturbance, and pain scores. Sociodemographic and disease-related characteristics were compared across classes.

**Findings:**

Among 2212 participants, we identified five classes, including four classes with “Low” (565 participants, 26%), “Normal” (651 participants, 29%), “High” (569 participants, 26%), or “Very High” (193 participants, 9%) symptom levels across all symptoms. Participants in a fifth class, “High Fatigue/Sleep/Pain and Low Anxiety/Depression” (234 participants, 11%) had similar levels of fatigue, sleep disturbance, and pain as in the “High” class but low anxiety and depression symptoms. There were significant and substantive trends in sociodemographic characteristics (age, education, race or ethnicity, marital or partner status) and increasing disease severity (diffuse disease, tendon friction rubs, joint contractures, gastrointestinal symptoms) across severity-based classes. Disease severity and sociodemographic characteristics of “High Fatigue/Sleep/Pain and Low Anxiety/Depression” class participants were similar to the “High” severity class.

**Interpretation:**

Most people with systemic sclerosis can be classified by levels of patient-reported symptoms, which are consistent across symptoms and highly associated with sociodemographic and disease-related variables, except for one group which reports low mental health symptoms despite high levels of other symptoms and substantial disease burden. Studies are needed to better understand resilience in systemic sclerosis and to identify and facilitate implementation of cognitive and behavioural strategies to improve coping and overall quality of life.

**Funding:**

10.13039/100000056National Institute of Nursing Research (F31NR019007), 10.13039/501100000024Canadian Institutes of Health Research, 10.13039/501100000142Arthritis Society Canada, the 10.13039/501100013455Lady Davis Institute for Medical Research, the 10.13039/501100020986Jewish General Hospital Foundation, 10.13039/100008582McGill University, 10.13039/501100019175Scleroderma Society of Ontario, Scleroderma Canada, Sclérodermie Québec, Scleroderma Manitoba, 10.13039/501100023335Scleroderma Atlantic, 10.13039/501100023446Scleroderma Association of BC, Scleroderma SASK, Scleroderma Australia, Scleroderma New South Wales, 10.13039/501100023332Scleroderma Victoria, and Scleroderma Queensland.

**Research in context**

**Evidence before this study**

We searched PubMed from database inception to December 7, 2022 using the terms “scleroderma” or “systemic sclerosis” with “symptom cluster” or “symptom clusters” or “co-occurring symptoms” or “concurrent symptoms” or “multiple symptoms” or “pain” or “fatigue” or “sleep disturbance” or “anxiety” or “depression” and “latent profile analysis” or “latent class analysis” or “cluster analysis.” We identified several studies that have examined patterns of single symptoms (e.g., pain) over time in systemic sclerosis or that have used latent profile analysis for other purposes but only one study that attempted to identify classes of co-occurring patient-reported symptom outcomes. That study, which was published in 2022, analysed data from 587 participants at a single United States academic medical centre and used cluster analysis to identify three subgroups, or classes, of people with “no/minimal,” “mild,” or “moderate” symptoms of dyspnoea, pain, fatigue, sleep disturbance, anxiety, and depression.

**Added value of this study**

This is the first study to fully capture the co-occurrence of common symptoms in systemic sclerosis and to identify a subgroup of people with the disease who appear to have avoided negative mental health outcomes that, for most patients, track closely with their disease severity and symptoms. We identified five distinct subgroups, or classes, of people with systemic sclerosis who shared similar symptom patterns of anxiety, depression, fatigue, sleep disturbance, and pain among 2212 adults with systemic sclerosis from 48 centres in seven countries. Four of the classes were based on increasing levels of symptom severity of self-reported anxiety, depression, fatigue, sleep disturbance, and pain from “Low” to “Normal” to “High” and “Very High,” and these levels were robustly associated with sociodemographic and disease-related variables. The other class, “High Fatigue/Sleep/Pain and Low Anxiety/Depression,” was similar to the “High” class in terms of self-reported and objective systemic sclerosis manifestations and sociodemographic characteristics, however, members of the class had low anxiety and depression symptoms.

**Implications of all the available evidence**

Resilience is defined as positive adjustment or the ability to preserve mental health in the context of adverse circumstances, and in many chronic diseases, people who exhibit resilient coping report lower levels of mental health concerns and higher quality of life compared to others with similar objective disease burden. Our study identified a class of people with systemic sclerosis who appear to cope well despite high levels of symptoms and overall disease burden. Resilience has not been studied in systemic sclerosis, and our study underlines a need for exploration of resilience and of cognitive and behavioural strategies that could lead to improved coping and quality of life in people with systemic sclerosis. Our results also underline the need, clinically, for comprehensive assessment in systemic sclerosis of co-occurring symptoms, which for most patients do not occur in isolation.

## Introduction

Systemic sclerosis is a chronic, progressive, autoimmune disease characterized by skin thickening and fibrosis of internal organs.^1^ There is no cure, and significant heterogeneity in disease progression and severity, organ involvement, and symptom manifestations make clinical management challenging.^2^ The symptom experience in systemic sclerosis is complex, but people with systemic sclerosis have emphasized the negative impacts of pain, fatigue, sleep disturbance, anxiety, and depression on physical function and quality of life.^3^, ^4^, ^5^, ^6^ Most research on these symptoms in systemic sclerosis, however, has examined only one symptom at a time without evaluating the degree to which symptoms co-occur or how sociodemographic and disease characteristics may be associated with patterns of co-occurring symptoms.^7^, ^8^, ^9^, ^10^, ^11^, ^12^, ^13^, ^14^

Symptom clusters in chronic diseases reflect groups of two or more symptoms that are related to one another, occur together, and are distinct from other symptom clusters.^15^ In systemic sclerosis, pain, fatigue, sleep disturbance, anxiety symptoms, and depression symptoms are highly associated^16^, ^17^, ^18^ and may synergistically influence quality of life.^18^ The only study that has examined clusters of co-occurring patient-reported symptoms in systemic sclerosis evaluated cross-sectional reports of pain, fatigue, sleep disturbance, depression symptoms, anxiety symptoms, and dyspnoea among 587 participants from a single centre in the United States.^19^ That study used cluster analyses to identify groups of participants with “no/minimal,” “mild,” or “moderate” symptoms, and reported that pain, fatigue, and sleep disturbance were predominant in the “mild” group, and pain, fatigue, sleep disturbance plus depression and anxiety symptoms were predominant in the “moderate” group. Associations of group membership with disease subtype and modified Rodnan skin scores were examined, but systemic sclerosis is a heterogenous disease, and no associations with specific disease manifestations (e.g., gastrointestinal symptoms, digital ulcers, joint contractures) were evaluated. Furthermore, although there is no fixed mechanism for determining the ideal sample size for cluster analysis or other methods for grouping symptom profiles, such as latent profile analysis,^20^, ^21^, ^22^ samples of even 500–600 participants often fail to extract clearly defined classes or all relevant classes,^21^^,^^23^ which may have been a limitation in that study.

A better understanding of patterns of co-occurring patient-reported symptoms in systemic sclerosis and their relationship with sociodemographic and disease-related characteristics would provide critical information to support investigation of symptom aetiology and coping and the development of tailored intervention approaches in a highly heterogenous disease. The objectives of our study were to investigate in a large international, multi-centre systemic sclerosis cohort of over 2200 participants from 48 centres: (1) classes of people with systemic sclerosis who share distinct symptom experiences within the symptom cluster of anxiety, depression, fatigue, sleep disturbance, and pain; and (2) examine patterns of sociodemographic and disease-related characteristics across classes.

## Methods

### Study design and participants

In this multi-centre cross-sectional study, we assessed baseline data collected at the time of enrolment in the ongoing Scleroderma Patient-centered Intervention Network (SPIN) Cohort.^24^ The SPIN Cohort sample is a convenience sample. Eligible participants at SPIN recruiting sites who are classified as having systemic sclerosis by a SPIN physician according to 2013 American College of Rheumatology/European League Against Rheumatism criteria,^25^ are 18 years of age or older, and are fluent in English, French, or Spanish are invited to participate by a physician or nurse coordinator.^24^ Written informed consent is obtained, and an online medical data form is completed by the SPIN physician or nurse coordinator to initiate registration. Participants then receive an email with instructions on how to complete patient-reported outcome measures online at enrolment and every three months thereafter. The SPIN Cohort study was approved by the Research Ethics Committee of the Centre intégré universitaire de santé et de services sociaux du Centre-Ouest-de-l'Île-de-Montréal (#MP-05-2013-150) and by the ethics committees of all recruiting sites. This study used baseline data collected between April 2014 and July 2020 from 48 SPIN centres in Australia, Canada, France, Mexico, Spain, the United Kingdom, and the United States of America. SPIN Cohort participants are comparable to those of other major systemic sclerosis cohorts.^26^ In the present study, data were included for participants who completed the Patient-Reported Outcomes Measurement Information System-29 version 2.0 (PROMIS-29 v2.0) measure^27^ at baseline and were not missing data for the anxiety, depression, fatigue, and sleep disturbance domains and the pain intensity item.

### Procedures and outcomes

SPIN physicians completed a medical data form which included participants’ age, sex, date of initial onset of non-Raynaud’s symptoms, date of diagnosis, systemic sclerosis subtype, and the presence of disease-related characteristics, including Raynaud’s phenomenon; modified Rodnan skin score^28^; distal digital tip ulcers (i.e., digital pulp, distal to distal interphalangeal joints); digital tip ulcers anywhere else on the fingers; current tendon friction ribs; small joint contractures (none to mild [defined as ≤25% range of motion limitation] or moderate to severe [defined as >25% range of motion limitation]); oesophageal involvement (dysphagia, heartburn, and/or reflux due to systemic sclerosis, now or in the past); stomach involvement (early satiety and/or vomiting due to systemic sclerosis, now or in the past); intestinal involvement (diarrhoea, bloating, and/or constipation due to systemic sclerosis, now or in the past); interstitial lung disease (seen on high-resolution computed tomography or chest radiography, or occurrence of “Velcro” crackles on auscultation, not due to another cause, now or in the past); and pulmonary arterial hypertension (diagnosed by right-sided heart catheterization according to standard definitions, now or in the past). All medical variables were assessed by physicians.

Participants reported sociodemographic information (including race or ethnicity) and completed patient-reported outcome measures. These measures were completed at the time the medical data form was completed. The PROMIS-29 v2.0 measure^27^ was used to assess the five symptoms in the symptom cluster. Domain scores were used for anxiety symptoms, depression symptoms, fatigue, and sleep disturbance. Raw subscale scores for the domains were derived by summing the four subscale item scores rated on a 5-point scale (1 = “not at all” to 5 = “very much”). Higher scores indicated greater symptom severity (e.g., more severe anxiety symptoms). The raw subscale scores (range 4–20) were converted to subscale T-scores standardized for the USA general population (mean = 50, standard deviation = 10).^29^ The reliability and validity of the PROMIS-29 measure have been demonstrated in systemic sclerosis.^30^, ^31^, ^32^ For anxiety, depression symptoms, fatigue, and sleep disturbance, mean T-scores at or near the following were used to differentiate the levels of symptom severity: <50 = low; 50 = normal to mild; 60 = high; and >60 = very high.

Pain intensity was measured using a single item from the PROMIS-29 v2.0 measure and was rated using an 11-point Likert scale ranging from 0 (no pain) to 10 (worst imaginable pain).^27^^,^^32^ A T-score was not available for pain intensity. Instead, pain thresholds previously used in systemic sclerosis^7^^,^^8^ guided interpretation of pain intensity scores: 0 = no pain, 1–4 = mild pain, 5–7 = moderate pain, and 8–10 = severe pain. We included pain intensity rather than the PROMIS-29 v2.0 pain interference domain score because our cluster was defined by symptoms that are experienced rather than the effects of those symptoms on daily activities or quality of life.

### Statistical analysis

Descriptive statistics were used to detail sociodemographic and disease-related characteristics and PROMIS-29 v2.0 domain raw scores and T-scores.

Latent profile analysis was used to identify homogenous subgroups, or latent classes, of participants who shared similar symptom patterns within the symptom cluster of anxiety symptoms, depression symptoms, fatigue, sleep disturbance, and pain. These symptoms were selected *a priori* as a cluster based on previous research in systemic sclerosis^16^, ^17^, ^18^ and other chronic autoimmune conditions^33^^,^^34^ that has found high levels of associations between the symptoms and synergistic effects on function and quality of life. Standard assumptions of latent profile analysis include local independence and equal error variances across classes.

Latent profile analysis models with different numbers of latent classes were tested to evaluate the optimal number of classes. Each model included T-scores for anxiety symptoms, depression symptoms, fatigue, and sleep disturbance and the single-item pain intensity score. To determine the best-fitting model and optimal number of classes, we referred to the Akaike Information Criteria (AIC), Bayesian Information Criteria (BIC), Bootstrapped Likelihood Ratio Test (BLRT), sample size adjusted Bayesian Information Criteria (ssBIC), and the Vuong-Lo-Mendell-Rubin likelihood ratio test (VLMR) model fit indicators. We sought to select a model that had relatively lower AIC, BIC, BLRT, ssBIC, VLMR,^21^ entropy level ≥0.80,^35^ and identified clinically meaningful classes.^36^ Additionally, we excluded models with class sizes <25 due to lack of parsimony and reduced power.^37^^,^^38^ Consistent with best practice recommendations, we followed a holistic approach which involved a series of decision steps, including evaluation of the goodness-of-fit indices for each model, entropy levels, and likelihood ratio tests to compare each candidate model with a model with one fewer class, and the overall plausibility of each model.^37^^,^^39^

We tested differences between classes for each sociodemographic and disease-related characteristic. We used one-way analysis of variance (ANOVA) for continuous variables and chi-square tests for categorical variables after ensuring that assumptions for homogeneity of variance were met for analysis of variance and minimum cell sizes for chi-square tests. Analysis of variance was performed using a General Linear Model approach due to unequal class sizes for continuous characteristics. The assumptions of the ANOVA, chi-square tests, and regression analyses were evaluated for each model, including normality and homogeneity of variance. Log-transformed values for time since diagnosis were used in the analyses because the variable was severely skewed and did not meet the ANOVA and regression assumptions prior to the transformation to normalize the data distribution. For the bivariate (simple linear) regression analyses, scatterplots and standardized residual plots were used to determine whether the assumption of linearity was met. Additionally, regression diagnostic procedures and standardized residual plots were used to determine whether the other assumptions of the bivariate regression model were met.

Since the latent profile analysis resulted in four classes that incrementally increased in symptom severity from low to very high, we performed post-hoc trend analyses to determine if sociodemographic and disease-related characteristics that differed between classes increased in severity or proportion with increasing class symptom severity. We used bivariate regression for continuous variables. Simple logistic regression was used in the bivariate analyses for dichotomous variables. Additionally, we illustrated the degree of differences across classes by comparing differences between the “Low” class and the “Very High” classes using mean differences with Wald 95% confidence intervals (CIs) for continuous variables and odds ratios with 95% CI for dichotomous variables. Likewise, we compared sociodemographic and disease-related characteristics in the “High Fatigue/Sleep/Pain and Low Anxiety/Depression” class with the “High” class given their similarities in all self-reported symptom domains except for anxiety and depression.

Mplus version 8.3 was used to conduct the latent profile analysis (Muthen & Muthen, Los Angeles, CA), and other analyses were done with SAS version 9.4 (SAS Institute, Cary, NC).

### Sample size

A sample size of 2212 adults with systemic sclerosis was sufficiently large and heterogenous to accurately extract up to eight subgroups based on five symptoms in the prespecified symptom cluster. The recommended sample size for latent profile analysis depends on several considerations, such as the number of classes expected to be extracted and the targeted margin of error.^37^ We were restricted to using participants with data from the SPIN Cohort, but all estimations of the number of participants needed for the number of classes and other characteristics of our models were substantially fewer than the number of participants included in our analyses.

### Role of the funding source

No study funder had any role in study design, data collection, data analysis, data interpretation, writing of the report, or the decision to submit for publication.

## Results

### Sample characteristics

**Fig. 1:**
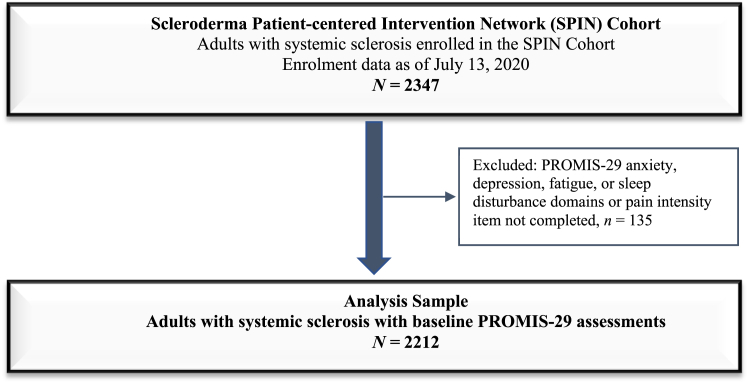
**Determination of the final analysissample.**

Of 2347 participants enrolled in the SPIN Cohort, 135 were missing one or more measures included in the symptom cluster analyses; therefore 2212 (95%) were included in analyses (Fig. 1). A sensitivity analysis did not reveal significant differences in the participant characteristics for those excluded when compared to those included in the analyses. Table 1 presents sociodemographic and disease-related characteristics. The mean age was 54.8 years (SD = 12.7; range = 18–89 years), and most of the sample was female (1939 [88%] participants), White (1834 [83%] participants), and married or living with a partner (1570 [71%] participants). The sample primarily included participants from the United States (778 [35%] participants), France (566 [26%] participants), and Canada (529 [24%] participants). The mean time since diagnosis was 9.4 years (SD = 8.1; range = 0–56 years). 856 (39%) participants had diffuse disease, and the most common disease-related characteristics included oesophageal symptoms (1855 [85%] participants), digital ulcers (857 [40%] participants), intestinal symptoms (836 [39%] participants), and interstitial lung disease (768 [36%] participants).

**Table 1:** Sample sociodemographic and disease characteristics (*N* = 2212).

Characteristics	n^a^	Mean (SD; range) or n (%)
**Sociodemographic**		
Age, years	2212	54.8 (12.7; 18–89)
Sex, female	2212	1939 (87.7%)
Race or ethnicity^b^	2210	
White		1834 (83.0%)
Black		144 (6.5%)
Other		232 (10.5%)
Country	2212	
United States		778 (35.2%)
France		566 (25.6%)
Canada		529 (23.9%)
United Kingdom		239 (10.8%)
Spain		40 (1.8%)
Australia		39 (1.8%)
Mexico		21 (1.0%)
Education, years	2208	14.8 (3.2; 8–20)
Married or living with partner	2212	1570 (71.0%)
**Systemic sclerosis**		
Time since diagnosis, years	2129	9.4 (8.1; 0–56)
Time since non-Raynaud’s symptom onset, years	2036	11.1 (8.7; 0–56)
Diffuse subtype	2190	856 (39.1%)
Raynaud’s phenomenon	2194	2152 (98.1%)
Modified Rodnan skin score (MRSS)	1615	8.8 (8.1; 1–48)
Digital ulcers	2169	857 (39.5%)
Tendon friction rubs	1946	451 (23.2%)
Moderate—severe small joint contractures	2092	541 (25.9%)
Moderate—severe large joint contractures	2049	257 (12.5%)
Esophageal symptoms	2181	1855 (85.1%)
Stomach symptoms	2130	643 (30.2%)
Intestinal symptoms	2156	836 (38.8%)
Interstitial lung disease	2161	768 (35.5%)
Pulmonary arterial hypertension	2092	186 (8.9%)

Variables in symptom cluster	n^a^	Mean (SD)^c^	Median (interquartile range)
Anxiety T-score	2212	52.2 (10.0)	53.7 (40.3–59.5)
Depression T-score	2212	51.4 (9.4)	51.8 (41.0–58.9)
Fatigue T-score	2212	54.9 (11.1)	55.1 (48.6–62.7)
Sleep disturbance T-score	2212	52.5 (8.6)	52.4 (46.2–57.9)
Pain intensity score (0–10 scale)	2212	3.6 (2.6)	3.0 (1.0–6.0)

SD = standard deviation.

a n indicates number of participants with data for variable.

b Race or ethnicity data were self-reported in each country using standard categories used in that country. Therefore, categories differed between countries.

c PROMIS-29 v2.0 T-scores standardized for the United States general population (mean = 50, SD = 10). T-scores were not available for the pain intensity item.

The PROMIS-29 v2.0 raw scores were determined for anxiety (mean = 7.6, SD = 3.7), depression symptoms (mean = 7.3, SD = 3.8), fatigue (mean = 11.4, SD = 4.7), and sleep disturbance (mean = 11.3, SD = 3.9). The mean raw scores were converted to mean T-scores for anxiety symptoms, depression symptoms, fatigue, and sleep disturbance. These T-scores ranged between 51.4 (SD = 9.4) and 54.9 (SD = 11.1). The mean pain intensity score was 3.6 (SD = 2.6). The PROMIS-29 v2.0 medians and interquartile ranges (IQR) for each symptom T-score were: anxiety (median = 53.7, IQR = 40.3–59.5), depression symptoms (median = 51.8, IQR = 41.0–58.9), fatigue (median = 55.1, IQR = 48.6–62.7), and sleep disturbance (median = 52.4, IQR = 46.2–59.9). The median pain intensity score was 3.0 (IQR = 1.0–6.0).

### Identification of latent classes

A model with five distinct classes was selected (see Table 2 for model fit indices) and was the most parsimonious and clinically relevant model. A 6-class model had minimally lower goodness of fit indices, but it included two very similar “High Fatigue/Sleep/Pain and Low Anxiety/Depression” classes that were not clinically distinguishable. For the selected latent profile analysis model, the entropy value was 0.885 and did identify distinct classes that were unique and clinically meaningful.

**Table 2:** Latent profile analysis: model fit information for the number of specified latent classes.

N classes	Number of parameters	Log-likelihood	AIC	BIC	ssBIC	Entropy	VLMR	BLRT	n (%) in smallest class
2	16	−41,329.445	82,690.890	82,782.117	82,731.282	0.831	p < 0.0001	p < 0.0001	992 (44.9)
3	22	−40,907.162	81,858.325	81,983.761	81,913.864	0.819	p = 0.0008	p < 0.0001	446 (20.2)
4	28	−40,630.925	81,317.849	81,477.496	81,388.535	0.802	p = 0.0003	p < 0.0001	330 (14.9)
5	34	−40,370.683	80,809.366	81,003.222	80,895.199	0.885	p < 0.0001	p < 0.0001	195 (8.8)
6	40	−40,235.250	80,550.500	80,778.566	80,651.480	0.863	p = 0.0002	p < 0.0001	188 (8.5)
7	46	−39,869.354	79,830.707	80,092.983	79,946.834	0.923	p < 0.0001	p < 0.0001	19 (0.9)
8	52	−39,741.213	79,586.426	79,882.912	79,717.700	0.906	p = 0.0061	p < 0.0001	19 (0.9)

AIC = Akaike Information Criteria; BIC = Bayesian Information Criteria; BLRT = parametric bootstrapped likelihood ratio test; ssBIC = sample size adjusted Bayesian Information Criteria; VLMR = Vuong-Lo-Mendell-Rubin likelihood ratio test.

**Fig. 2:**
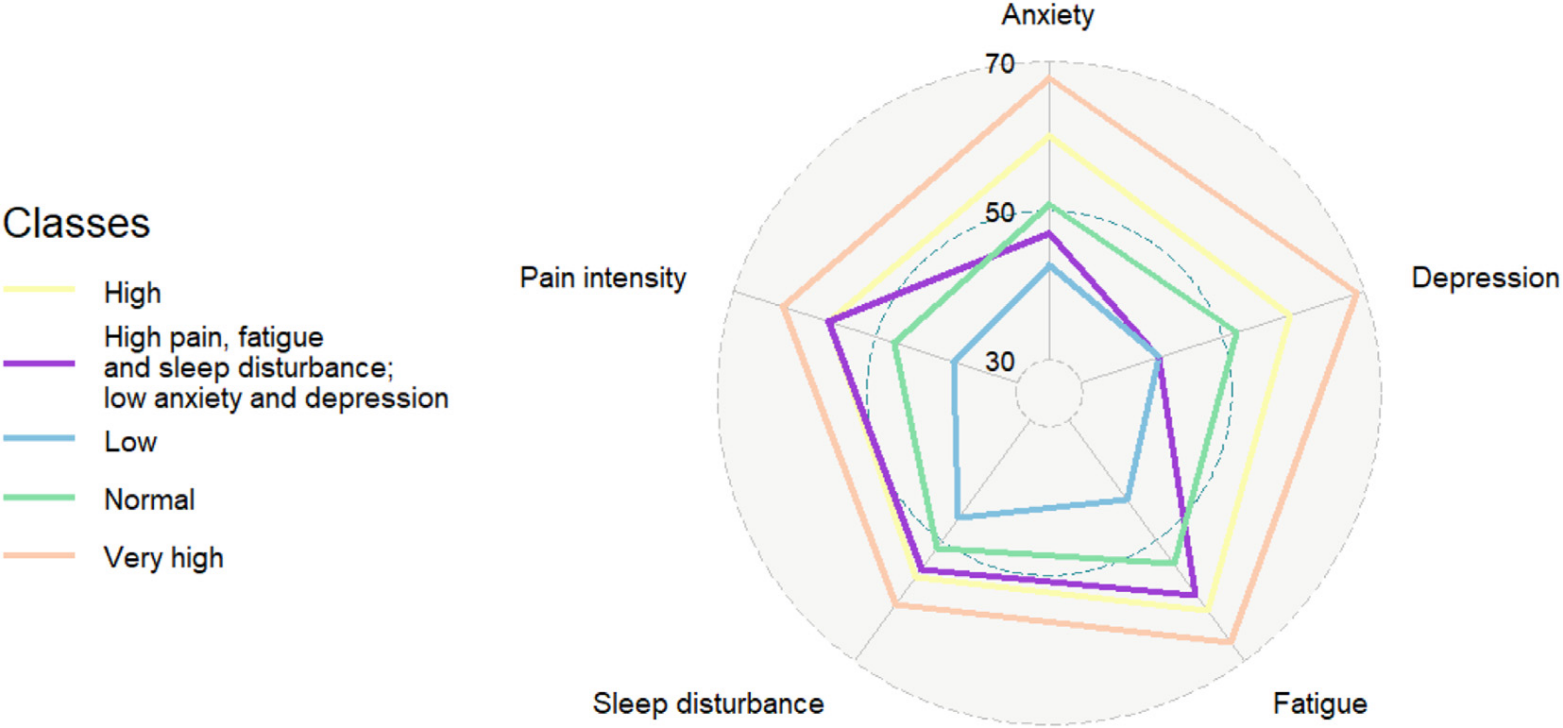
**Means scores for each domain in the five classes**. T-scores from the PROMIS-29 measure for anxiety, depression, fatigue, and sleep disturbance domains were used. Pain intensity item scores were converted to T-scores based on our study sample mean and standard deviation.

Table 3 provides an overview of the five classes and their symptom scores. The “Low” class (565 [26%] participants) was characterized by T-scores notably lower than the USA general population mean (T-scores ≤46.3) and pain intensity scores on the low end of the mild range (mean = 1.4). The “Normal” class (651 [29%] participants) had T-scores similar to the population mean (at or near 50) with mild pain (mean = 3.1). The “High” class (569 [26%] participants) had mean domain scores between 6.1 and 11.7 points greater than the population mean and low to moderate pain (mean = 5.0). The “Very High” class (193 [9%] participants) had the most severe levels of all five symptoms with mean domain scores 10.7–19.0 points above the population level and moderate pain (mean = 6.3). The “High Fatigue/Sleep/Pain and Low Anxiety/Depression” class (234 [11%] participants) was characterized by levels of fatigue, sleep disturbance, and pain similar to the “High” class but low levels of anxiety and depression symptoms. Fig. 2 presents a spider plot that illustrates the pattern of scores for each symptom across classes.

**Table 3:** Classes and their domain T-scores (anxiety, depression, fatigue, sleep disturbance) or single-item (pain intensity) scores (*N* = 2212).^a^

Symptoms	Low (n = 565, 26%)	Normal (n = 651, 29%)	High (n = 569, 26%)	Very high (n = 193, 9%)	High fatigue/sleep/pain Low anxiety/depression (n = 234, 11%)
Anxiety	42.7 (4.9)	50.8 (6.9)	60.1 (5.4)	67.7 (5.7)	47.0 (7.3)
Depression	41.1 (0.8)	52.0 (2.7)	59.4 (2.8)	69.0 (3.9)	41.0 (0.0)
Fatigue	43.3 (7.5)	53.9 (8.6)	61.7 (7.8)	66.9 (6.6)	59.1 (7.3)
Sleep disturbance	46.3 (7.2)	51.3 (7.8)	56.1 (7.0)	60.7 (7.2)	54.7 (7.6)
Pain intensity	1.4 (1.5)	3.1 (2.2)	5.0 (2.3)	6.3 (2.1)	5.0 (2.0)

SD = standard deviation.

a T-scores standardized for the United States general population (mean = 50, SD = 10) for anxiety, depression, fatigue, and sleep disturbance. T-scores are not available for the pain intensity item (possible range 0–10 with higher scores representing greater pain intensity).

### Comparisons of sociodemographic and disease-related characteristics by classes

Table 4 shows sociodemographic and disease-related characteristics of participants in each class. There were statistically significant differences between classes for four sociodemographic (age, years of education, married or living with partner, White vs. Black or other race or ethnicity) and seven disease-related characteristics (diffuse disease, tendon friction rubs, moderate to severe small joint contractures, moderate to severe large joint contractures, oesophageal symptoms, stomach symptoms, intestinal symptoms).

**Table 4:** Class differences in sociodemographic and disease-related characteristics (*N* = 2212).

Characteristics	Total n	Low (n = 565, 25.5%) Mean (SD) or n (%)	Normal (n = 651, 29.4%) Mean (SD) or n (%)	High (n = 569, 25.7%) Mean (SD) or n (%)	Very high (n = 193, 8.7%) Mean (SD) or n (%)	High fatigue/sleep/pain Low anxiety/depression (n = 234, 10.6%) Mean (SD) or n (%)	p-value for differences by class
**Sociodemographic**							
Age, in years	2212	56.6 (12.4)	55.5 (12.9)	53.2 (12.2)	51.0 (12.2)	55.1 (13.1)	<0.0001
Years of education	2208	15.1 (3.2)	15.0 (3.2)	14.6 (3.2)	14.2 (3.3)	14.8 (3.0)	0.0017
Sex, female	2212	482 (85.3)	569 (87.4)	507 (89.1)	173 (89.6)	208 (88.9)	0.2828
Race/ethnicity	2210						
White race/ethnicity		490 (86.7)	535 (82.4)	459 (80.7)	153 (79.3)	197 (84.2)	0.0400^a^
Black race/ethnicity		28 (5.0)	41 (6.3)	43 (7.6)	15 (7.8)	17 (7.3)	
Other race/ethnicity		47 (8.3)	73 (11.3)	67 (11.8)	25 (13.0)	20 (8.6)	
Married/living with partner	2212	422 (74.7)	472 (72.5)	388 (68.2)	126 (65.3)	162 (69.2)	0.0397
**Disease-related**							
Time since diagnosis, in years	2129	9.8 (8.4)	9.3 (7.6)	8.9 (7.9)	8.8 (7.7)	10.4 (9.1)	0.6740
Time since diagnosis, in year (log)	2129	2.1 (0.8)	2.0 (0.8)	2.0 (0.9)	2.0 (0.8)	2.1 (0.9)	0.1153
Diffuse subtype	2190	197 (35.1)	260 (40.2)	228 (40.6)	88 (46.8)	83 (35.6)	0.0329
Digital ulcers	2169	215 (38.7)	236 (37.1)	232 (41.4)	84 (44.7)	90 (39.3)	0.3170
Tendon friction rubs	1946	87 (16.7)	139 (24.2)	118 (23.9)	46 (29.9)	61 (30.1)	0.0002
Moderate-severe small joint contractures	2092	104 (19.3)	145 (23.5)	164 (30.4)	67 (37.2)	61 (28.2)	<0.0001
Moderate-severe large joint contractures	2049	50 (9.5)	72 (11.8)	69 (13.1)	36 (20.7)	30 (14.0)	0.0033
Esophageal symptoms	2181	439 (78.1)	543 (84.6)	489 (87.8)	176 (93.1)	208 (90.0)	<0.0001
Stomach symptoms	2130	116 (21.4)	1.79 (28.5)	200 (36.8)	72 (38.5)	76 (33.3)	<0.0001
Intestinal symptoms	2156	152 (27.8)	236 (37.0)	249 (45.0)	90 (47.9)	109 (47.2)	<0.0001

a Chi-square test of White vs. Black or other race or ethnicity.

All variables with statistically significant class differences (Table 4) also had statistically significant trends from Low to Normal to High to Very High classes. Table 5 illustrates the magnitude of differences for class comparisons between the “Low” and “Very High” classes across the trend.

**Table 5:** Trend analysis and class comparisons for sociodemographic and disease-related characteristics among variables with class differences.

Characteristics	Trend (Low to normal to high to very high classes) p-value	Low vs. very high	High fatigue/sleep/pain and low anxiety/depression vs. high
**Continuous variables**		**Mean difference (95% CI)**	**Mean difference (95% CI)**
Age, in years	<0.0001	5.59 (3.57, 7.62)	1.92 (0.02, 3.83)
Years of education	<0.0001	0.89 (0.37, 1.42)	0.21 (−0.27, 0.69)
**Dichotomous variables**		**OR (95% CI)**	**OR (95% CI)**
White race/ethnicity	0.0029	0.84 (0.73, 0.96)	0.92 (0.85, 1.01)
Married/living with partner	0.0021	0.86 (0.77, 0.97)	0.95 (0.69, 1.32)
Diffuse subtype	0.0049	1.18 (1.05, 1.32)	1.24 (0.90, 1.70)
Tendon friction rubs	0.0003	1.29 (1.12, 1.48)	0.73 (0.51, 1.06)
Moderate-severe small joint contractures	<0.0001	1.35 (1.20, 1.53)	1.11 (0.78, 1.57)
Moderate-severe large joint contractures	0.0004	1.35 (1.16, 1.58)	0.93 (0.59, 1.48)
Esophageal symptoms	<0.0001	1.56 (1.28, 1.90)	0.80 (0.48, 1.31)
Stomach symptoms	<0.0001	1.32 (1.17, 1.49)	1.16 (0.84, 1.61)
Intestinal symptoms	<0.0001	1.34 (1.19, 1.50)	0.92 (0.67, 1.25)

CI = confidence interval; OR = odds ratio.

Table 5 also compares sociodemographic and disease characteristics between the “High” class and the “High Fatigue/Sleep/Pain and Low Anxiety/Depression” class. They were similar, and there were no statistically significant differences for any disease-related characteristics. The only statistically significant difference was that members of the class with low anxiety and depression symptoms were approximately two years older on average than members of the “High” class.

## Discussion

In this study, we identified five homogenous subgroups of people with systemic sclerosis who shared distinct symptom experiences of anxiety, depression, fatigue, sleep disturbance, and pain within the symptom cluster. We identified a trend among four of the subgroups in which increasing symptom severity from Low to Normal to High to Very High was robustly associated with sociodemographic and disease-related characteristics, including decreasing age, fewer years of education, less likelihood of being married or living with a partner, greater likelihood of self-reported non-White race or ethnicity, and the presence of more severe disease, including higher likelihood of having diffuse disease, tendon friction rubs, moderate to severe small joint contractures, moderate to severe large joint contractures, oesophageal symptoms, stomach symptoms, and intestinal symptoms. The fifth subgroup differed markedly from the others in that individuals experienced high levels of fatigue, sleep disturbance, and pain but low levels of anxiety and depression symptoms. This subgroup was similar in all sociodemographic and disease-related characteristics to the “High” symptom subgroup, except for being approximately two years older.

This trend in symptom severity among subgroups is generally congruent with the only previous study of symptom patterns in systemic sclerosis, which explored patterns of pain, fatigue, sleep disturbance, anxiety, depression, and dyspnoea in a single-centre study with 587 participants.^19^ However, patterns were less clearly demarcated in that study compared to our study, which may be due to the smaller number of participants. In that study, “no/minimal,” “mild,” and “moderate” symptom subgroups were identified, but rather than congruent level increases across classes, the “mild” symptom subgroup included those in which pain, fatigue, and sleep disturbance were predominant, whereas the “moderate” symptom subgroup included participants with higher severity scores for pain, fatigue, sleep disturbance, anxiety symptoms, and depression symptoms. Furthermore, there were no differences in global disease severity indicators, including diffuse disease subtype, skin scores, and disease duration between the “mild” and “moderate” classes, and associations with more specific disease manifestations (e.g., gastrointestinal symptoms, digital ulcers, joint contractures, tendon friction rubs) were not evaluated.

In this study, we identified a unique class characterized by high fatigue, sleep disturbance, and pain, but low anxiety and depression symptoms. There were no substantive differences between sociodemographic and disease-related variables between this subgroup and the “High” symptom class. Members of both classes experienced substantial disease burden and similar fatigue, sleep disturbance, and pain; however, this group had very low levels of anxiety and depression symptoms. These differences may be explained by resilience and how different people cope with the very high level of burden they face from their disease. Resilience is defined as positive adjustment or the ability to preserve mental health in the context of adverse circumstances.^40^^,^^41^ Psychological factors associated with resilience include self-efficacy, self-esteem, optimism, hardiness, determination, an internal locus of control, and a sense of self-empowerment and mastery.^42^ Systematic reviews have found that people with serious medical conditions who score higher on measures of resilience also report less anxiety and depression and greater quality of life.^42^^,^^43^ Interventions that include resilience-improving strategies and adaptive coping strategies have been effective at improving psychological adaptation and symptom burden in other chronic conditions,^44^, ^45^, ^46^ yet little is known about their impact on symptom severity and mental health in systemic sclerosis. No resilience measurement tools have been validated and no studies have been conducted on resilience in people with systemic sclerosis. A scale to measure resilience should be validated in systemic sclerosis, and patterns of symptoms should be examined in relation to resilience, particularly differences in resilience levels among various subgroups. Additionally, qualitative research with patients may be helpful to understand the role of resilience in positive mental health in systemic sclerosis.

Our findings underscore the need for research to better understand the characteristics of people with systemic sclerosis who are highly resilient, including validation of measurement tools and studies that compare characteristics of less and more resilient people. Ideally, programs to support resilient coping will be adapted from other conditions, tested, and made available to people with systemic sclerosis. Most symptom management interventions target individual symptoms, such as pain or depression, independently. Cognitive behavioural symptom-cluster interventions and psychoeducational interventions targeting co-occurring symptoms of fatigue, sleep disturbance, and pain have effectively reduced symptom burden in cancer patients.^47^, ^48^, ^49^ The development and testing of tailored symptom management interventions such as a multi-symptom intervention targeting a specific symptom cluster or a single intervention that is effective across multiple clustered symptoms in systemic sclerosis is needed.

Clinically, our results suggest the need for comprehensive assessment of symptoms to identify multiple co-occurring symptoms and symptoms that might have otherwise been overlooked. For most patients, changes in pain severity do not occur in isolation from changes in fatigue, sleep disturbance, anxiety, or depression symptoms, for instance. Previous research has emphasized the importance of disease-specific patient-reported measures to capture the patients’ experience with systemic sclerosis.^50^ However, current measures only evaluate single symptoms or symptoms as independent entities. To our knowledge, no measurement tools examine co-occurring symptoms, or symptom clusters, in people with systemic sclerosis or other rheumatic diseases. Additionally, current measurement tools for anxiety, depression, fatigue, sleep disturbance, and pain do not evaluate the multi-dimensional aspects of these symptoms such as their patterns (e.g., cyclical or chronic), fluctuations, duration, or the presence of a “trigger” symptom that serves as a driving factor for other symptoms.^51^ The development and testing of patient-reported measures that evaluate the co-occurrence and multi-dimensional aspects of these symptoms is needed to improve clinical management and quality of life in systemic sclerosis.

Our study had several strengths, including a large multinational sample that allowed for the use of a statistically robust analysis method, exploration of multiple covariates that have the potential to serve as predictors for symptom severity, and the use of a standardized, well-characterized measure to allow for comparison of subgroups across populations. There were also several limitations. While the SPIN Cohort is comparable to other large systemic sclerosis cohorts,^26^ we acknowledge that the SPIN Cohort is a convenience sample of participants with access to the internet and specialized systemic sclerosis care, which may limit the generalizability of our findings. We also recognize that socioeconomic status data were not available for SPIN Cohort participants, and that additional factors (e.g., co-existing fibromyalgia or arthritis) that may contribute to the symptom outcomes were not collected. As a largely descriptive study, we did not correct for multiple hypothesis testing, and we used cross-sectional data which provided only a single snapshot of participants’ symptoms. While the criteria used for pain severity was based on previously established cut-off scores, we recognize that this might not accurately reflect how individuals experience their symptoms. We also recognize that the lack of granularity for race and ethnicity is a limitation when trying to explore the effect of these variables on patient-reported outcomes in more detail. SPIN does collect more granular race and ethnicity data than the groups used in this manuscript. For each of the seven countries involved, however, multiple response option categories are used, and categories in one country are not the same and not recognized by participants in others. These disparities meant that the most granular data for race or ethnicity we could reasonably classify across countries was White, Black, and Other, and hence these categories were used in this study.

In summary, even though systemic sclerosis is a highly heterogeneous disease, we identified five homogenous subgroups, or classes, of individuals who shared similar symptom experiences with anxiety, depression, fatigue, sleep disturbance, and pain. We identified a trend in which worsening symptom severity across these classes corresponded closely with worse disease, based on overarching severity (disease subtype) and more specific manifestations (e.g., tendon friction rubs, joint contractures, gastrointestinal symptoms). However, there was one marked exception; members of one class had high levels of fatigue, sleep disturbance, and pain symptom severity and high levels of underlying disease severity but low anxiety and depression symptoms. More research is needed to better understand the potential contribution of resilience and adaptive coping to the experience of living with systemic sclerosis, as well as the multi-dimensional aspects of symptoms in this population. Clinically, healthcare providers should partner with patients to address symptoms of anxiety, depression, fatigue, sleep disturbance, and pain, including identifying sociodemographic and disease-related characteristics that are associated with more severe symptoms and implementing symptom management interventions to reduce symptom burden and improve quality of life.

## Contributors

RKW, MRK, DEB, TJS, LK, BDT, and SGS contributed to the conception and design of the study. LK, MEC, WRN, SJB, VLM, MH, and LM contributed to the oversight and management of data collection in the SPIN Cohort via roles on the SPIN Steering Committee. RKW, LK, BL, AB, BDT, and SGS contributed to data analysis, and accessed and verified the data. RKW, MRK, DEB, TJS, LK, BDT, and SGS contributed to the interpretation of results. RKW, MRK, DEB, TJS, BDT, and SGS drafted the manuscript. All authors provided critical review of the manuscript and approved the final manuscript. RKW was the guarantor.

## Data sharing statement

De-identified individual participant data with a data dictionary and analysis codes that were used to generate the results reported in this article will be made available upon request to the corresponding author and after presentation of a methodologically sound proposal that is approved by the Scleroderma Patient-centered Intervention Network Data Access and Publications Committee. Data will be available beginning 12 months after publication. Data requestors will need to sign a data transfer agreement.

## Declaration of interests

All authors declare no competing interests.
