# Supplementary material for: Patterns of patient-reported symptoms and association with sociodemographic and systemic sclerosis disease characteristics: a scleroderma Patient-centered Intervention Network (SPIN) Cohort cross-sectional study
Source: eClinicalMedicine. 2023 Jul 20;62:102104. doi: 10.1016/j.eclinm.2023.102104 (PMC10393558; doi:10.1016/j.eclinm.2023.102104)
Supplement: Supplemental File [file mmc1.docx]

**Supplemental File**

The SPIN Investigators include: Claire E. Adams, Jewish General Hospital, Montreal, Quebec, Canada; Richard S. Henry, Jewish General Hospital, Montreal, Quebec, Canada; Catherine Fortuné, Ottawa Scleroderma Support Group, Ottawa, Ontario, Canada; Karen Gottesman, National Scleroderma Foundation, Los Angeles, California, USA; Geneviève Guillot, Sclérodermie Québec, Longueuil, Quebec, Canada; Laura K. Hummers, Johns Hopkins University School of Medicine, Baltimore, Maryland, USA; Amanda Lawrie-Jones, Scleroderma Australia and Scleroderma Victoria, Melbourne, Victoria, Australia; Maureen D. Mayes, University of Texas McGovern School of Medicine, Houston, Texas, USA; Michelle Richard, Scleroderma Atlantic, Halifax, Nova Scotia, Canada; Maureen Sauvé, Scleroderma Society of Ontario, Hamilton, Ontario, Canada; Shervin Assassi, University of Texas McGovern School of Medicine, Houston, Texas, USA; Ghassan El-Baalbaki, Université du Québec à Montréal, Montreal, Quebec, Canada; Kim Fligelstone, Scleroderma & Raynaud’s UK, London, UK; Tracy Frech, University of Utah, Salt Lake City, Utah, USA; Amy Gietzen, National Scleroderma Foundation, Tri-State Chapter, Binghamton, New York, USA; Daphna Harel, New York University, New York, New York, USA; Monique Hinchcliff, Yale School of Medicine, New Haven, Connecticut, USA; Sindhu R. Johnson, Toronto Scleroderma Program, Mount Sinai Hospital, Toronto Western Hospital, and University of Toronto, Toronto, Ontario, Canada; Maggie Larche, McMaster University, Hamilton, Ontario, Canada; Catarina Leite, University of Minho, Braga, Portugal; Christelle Nguyen, Université Paris Descartes, Université de Paris, Paris, France, and Assistance Publique - Hôpitaux de Paris, Paris, France; Karen Nielsen, Scleroderma Society of Ontario, Hamilton, Ontario, Canada; Janet Pope, University of Western Ontario, London, Ontario, Canada; François Rannou, Université Paris Descartes, Université de Paris, Paris, France, and Assistance Publique - Hôpitaux de Paris, Paris, France; Tatiana Sofia Rodriguez-Reyna, Instituto Nacional de Ciencias Médicas y Nutrición Salvador Zubirán, Mexico City, Mexico; Anne A. Schouffoer, Leiden University Medical Center, Leiden, the Netherlands; Maria E. Suarez-Almazor, University of Texas MD Anderson Cancer Center, Houston, Texas, USA; Christian Agard, Centre Hospitalier Universitaire - Hôtel-Dieu de Nantes, Nantes, France; Nassim Ait Abdallah, Assistance Publique - Hôpitaux de Paris, Hôpital St-Louis, Paris, France; Marc André, Centre Hospitalier Universitaire Gabriel-Montpied, Clermont-Ferrand, France; Elana J. Bernstein, Columbia University, New York, New York, USA; Sabine Berthier, Centre Hospitalier Universitaire Dijon Bourgogne, Dijon, France; Lyne Bissonnette, Université de Sherbrooke, Sherbrooke, Quebec, Canada; Alessandra Bruns, Université de Sherbrooke, Sherbrooke, Quebec, Canada; Patricia Carreira, Servicio de Reumatologia del Hospital 12 de Octubre, Madrid, Spain; Marion Casadevall, Assistance Publique - Hôpitaux de Paris, Hôpital Cochin, Paris, France; Benjamin Chaigne, Assistance Publique - Hôpitaux de Paris, Hôpital Cochin, Paris, France; Lorinda Chung, Stanford University, Stanford, California, USA; Benjamin Crichi, Assistance Publique - Hôpitaux de Paris, Hôpital St-Louis, Paris, France; Christopher Denton, Royal Free London Hospital, London, UK; Robyn Domsic, University of Pittsburgh, Pittsburgh, Pennsylvania, USA; James V. Dunne, St. Paul's Hospital and University of British Columbia, Vancouver, British Columbia, Canada; Bertrand Dunogue, Assistance Publique - Hôpitaux de Paris, Hôpital Cochin, Paris, France; Regina Fare, Servicio de Reumatologia del Hospital 12 de Octubre, Madrid, Spain; Dominique Farge-Bancel, Assistance Publique - Hôpitaux de Paris, Hôpital St-Louis, Paris, France; Paul R. Fortin, CHU de Québec - Université Laval, Quebec, Quebec, Canada; Jessica Gordon, Hospital for Special Surgery, New York City, New York, USA; Brigitte Granel-Rey, Aix Marseille Université, and Assistance Publique - Hôpitaux de Marseille, Hôpital Nord, Marseille, France; Aurélien Guffroy, Les Hôpitaux Universitaires de Strasbourg, Nouvel Hôpital Civil, Strasbourg, France; Genevieve Gyger, Jewish General Hospital and McGill University, Montreal, Quebec, Canada; Eric Hachulla, Centre Hospitalier Régional Universitaire de Lille, Hôpital Claude Huriez, Lille, France; Sabrina Hoa, Centre hospitalier de l’Université de Montréal – CHUM, Montreal, Quebec, Canada; Alena Ikic, CHU de Québec - Université Laval, Quebec, Quebec; Niall Jones, University of Alberta, Edmonton, Alberta, Canada; Suzanne Kafaja, University of California, Los Angeles, California, USA; Nader Khalidi, McMaster University, Hamilton, Ontario, Canada; Kimberly Lakin, Hospital for Special Surgery, New York City, New York, USA; Marc Lambert, Centre Hospitalier Régional Universitaire de Lille, Hôpital Claude Huriez, Lille, France; David Launay, Centre Hospitalier Régional Universitaire de Lille, Hôpital Claude Huriez, Lille, France; Yvonne C. Lee, Northwestern University, Chicago, Illinois, USA; Hélène Maillard, Centre Hospitalier Régional Universitaire de Lille, Hôpital Claude Huriez, Lille, France; Nancy Maltez, University of Ottawa, Ottawa, Ontario, Canada; Joanne Manning, Salford Royal NHS Foundation Trust, Salford, UK; Isabelle Marie, CHU Rouen, Hôpital de Bois-Guillaume, Rouen, France; Maria Martin Lopez, Servicio de Reumatologia del Hospital 12 de Octubre, Madrid, Spain; Thierry Martin, Les Hôpitaux Universitaires de Strasbourg, Nouvel Hôpital Civil, Strasbourg, France; Ariel Masetto, Université de Sherbrooke, Sherbrooke, Quebec, Canada; François Maurier, Uneos - Groupe hospitalier associatif, Metz, France; Arsene Mekinian, Assistance Publique - Hôpitaux de Paris, Hôpital St-Antoine, Paris, France; Sheila Melchor Díaz, Servicio de Reumatologia del Hospital 12 de Octubre, Madrid, Spain; Mandana Nikpour, St Vincent’s Hospital and University of Melbourne, Melbourne, Victoria, Australia; Louis Olagne, Centre Hospitalier Universitaire Gabriel-Montpied, Clermont-Ferrand, France; Vincent Poindron, Les Hôpitaux Universitaires de Strasbourg, Nouvel Hôpital Civil, Strasbourg, France; Susanna Proudman, Royal Adelaide Hospital and University of Adelaide, Adelaide, South Australia, Australia; Alexis Régent, Assistance Publique - Hôpitaux de Paris, Hôpital Cochin, Paris, France; Sébastien Rivière, Assistance Publique - Hôpitaux de Paris, Hôpital St-Antoine, Paris, France; David Robinson, University of Manitoba, Winnipeg, Manitoba, Canada; Esther Rodríguez Almazar, Servicio de Reumatologia del Hospital 12 de Octubre, Madrid, Spain; Sophie Roux, Université de Sherbrooke, Sherbrooke, Quebec, Canada; Perrine Smets, Centre Hospitalier Universitaire Gabriel-Montpied, Clermont-Ferrand, France; Vincent Sobanski, Centre Hospitalier Régional Universitaire de Lille, Hôpital Claude Huriez, Lille, France; Robert Spiera, Hospital for Special Surgery, New York City, New York, USA; Virginia Steen, Georgetown University, Washington, DC, USA; Evelyn Sutton, Dalhousie University, Halifax, Nova Scotia, Canada; Carter Thorne, Southlake Regional Health Centre, Newmarket; John Varga, University of Michigan, Ann Arbor, Michigan, USA; Pearce Wilcox, St. Paul's Hospital and University of British Columbia, Vancouver, British Columbia, Canada; Mara Cañedo Ayala, Jewish General Hospital, Montreal, Quebec, Canada; Vanessa Cook, Jewish General Hospital, Montreal, Quebec, Canada; Sophie Hu, Jewish General Hospital, Montreal, Quebec, Canada; Bianca Matthews, Jewish General Hospital, Montreal, Quebec, Canada; Elsa-Lynn Nassar, Jewish General Hospital, Montreal, Quebec, Canada; Marieke Alexandra Neyer, Jewish General Hospital, Montreal, Quebec, Canada; Julia Nordlund, Jewish General Hospital, Montreal, Quebec, Canada; Sabrina Provencher, Jewish General Hospital, Montreal, Quebec, Canada.
